# A machine learning-based model for predicting the risk of early-stage inguinal lymph node metastases in patients with squamous cell carcinoma of the penis

**DOI:** 10.3389/fsurg.2023.1095545

**Published:** 2023-03-17

**Authors:** Li Ding, Chi Zhang, Kun Wang, Yang Zhang, Chuang Wu, Wentao Xia, Shuaishuai Li, Wang Li, Junqi Wang

**Affiliations:** Department of Urology, the Affiliated Hospital of Xuzhou Medical University, Xuzhou, China

**Keywords:** machine learning algorithms, prediction model, penis cancer, squamous cell carcinoma, inguinal lymph node metastases, real-world research

## Abstract

**Objective:**

Inguinal lymph node metastasis (ILNM) is significantly associated with poor prognosis in patients with squamous cell carcinoma of the penis (SCCP). Patient prognosis could be improved if the probability of ILNM incidence could be accurately predicted at an early stage. We developed a predictive model based on machine learning combined with big data to achieve this.

**Methods:**

Data of patients diagnosed with SCCP were obtained from the Surveillance, Epidemiology, and End Results Program Research Data. By combing variables that represented the patients' clinical characteristics, we applied five machine learning algorithms to create predictive models based on logistic regression, eXtreme Gradient Boosting, Random Forest, Support Vector Machine, and k-Nearest Neighbor. Model performance was evaluated by ten-fold cross-validation receiver operating characteristic curves, which were used to calculate the area under the curve of the five models for predictive accuracy. Decision curve analysis was conducted to estimate the clinical utility of the models. An external validation cohort of 74 SCCP patients was selected from the Affiliated Hospital of Xuzhou Medical University (February 2008 to March 2021).

**Results:**

A total of 1,056 patients with SCCP from the SEER database were enrolled as the training cohort, of which 164 (15.5%) developed early-stage ILNM. In the external validation cohort, 16.2% of patients developed early-stage ILNM. Multivariate logistic regression showed that tumor grade, inguinal lymph node dissection, radiotherapy, and chemotherapy were independent predictors of early-stage ILNM risk. The model based on the eXtreme Gradient Boosting algorithm showed stable and efficient prediction performance in both the training and external validation groups.

**Conclusion:**

The ML model based on the XGB algorithm has high predictive effectiveness and may be used to predict early-stage ILNM risk in SCCP patients. Therefore, it may show promise in clinical decision-making.

## Introduction

Penile carcinoma (PC) is a rare malignant tumor of the genitourinary system that exhibits a predilection for the glans and inner prepuce (1). The overall incidence of PC in men in developed countries such as Europe and the United States is less than 1 in 100,000, and it accounts for approximately 0.24% of all new cancer cases in men (2, 3). In contrast, PC accounts for 2% of malignancies in men in less developed countries or regions such as Africa and South America, and in some places like Uganda, the incidence is even higher than 8/100,000 (4, 5). This phenomenon could be linked to risk factors such as poor economic conditions, a high prevalence of HPV infection, and phimosis (6, 7). Squamous cell carcinoma accounts for more than 95% of penile cancers and is the most common histologic type (8). The risk of developing squamous cell carcinoma of the penis (SCCP) increases with age, with the peak age range being 50 to 70 years (9). For the surgical treatment of SCCP, the aim of primary tumor treatment is to completely remove the tumor and preserve as many organs as possible without compromising control of the tumor. However, for advanced invasive tumors, partial or radical penectomy is unavoidable (10).

Lymph node invasion of SCCP conforms to the anatomy, and it most commonly metastasizes to the inguinal lymph nodes, followed by the pelvic lymph nodes. Superficial and deep inguinal lymph nodes are the first regional lymph nodes to be invaded, most commonly in the superior medial region, either unilaterally or bilaterally (11). Previous studies have shown that the occurrence of lymph node metastases in SCCP patients is significantly associated with poor prognosis (12). The survival rate of patients with SCCP decreases dramatically with increase in lymph node invasion. The five-year survival rate of patients with inguinal lymph node metastasis (ILNM) is only 50%–80%, but the five-year survival rate of patients who develop pelvic lymph node and peripheral lymph node invasion is less than 33% (13, 14). Due to limitations in imaging techniques, up to 25% of cases in patients with clinically negative lymph nodes cannot be detected despite the presence of micrometastases (15). Therefore, identifying independent risk factors for the development of ILNM at an early stage and establishing corresponding risk prediction models for identifying patients at high risk of ILNM can improve the survival prognosis of this group of SCCP patients through more frequent imaging and more comprehensive clinical treatment in the early stage of diagnosis.

Early studies had shown that stage and grade of the tumor as well as lymphatic and vascular embolization were associated with the risk of lymph node metastasis (16, 17). Ficarra et al. showed that pT stage, histologic grade, venous embolization, and lymphatic embolization were independent predictors of lymph node metastasis in SCCP (18). Peak et al. developed a predictive model based on the clinicopathological characteristics of patients recorded in the National Cancer Database (NCDB), which showed better efficacy in their internal validation (19). In previous studies, several prediction nomograms were established based on logistic regression. The traditional logistic regression algorithm has some limitations in terms of nonlinear complex computation, with area under the curve (AUC) both less than 0.8, so this leaves room for further optimization (20, 21). Machine learning (ML) is an advanced algorithmic model that automatically learns and improves performance by identifying complex nonlinear relationships in different patterns, and is considered superior to traditional algorithms (22). As one of the components of artificial intelligence, ML has been widely used in clinical practice, such as for epidemic prediction (23, 24), and survival analysis (25, 26). ML models have also been proposed for the prediction of lymph node metastasis in a variety of malignancies (27, 28). Based on these promising clinical applications of ML models, the present study aimed to develop and validate a novel ML-based model for predicting the risk of early-stage ILNM in patients with SCCP.

## Materials and methods

### Data source and study population

A retrospective cohort design was adopted. Data were obtained from the SEER research database, which covers roughly 27.8% of the US population. The SEER database is a comprehensive data source, which is public and identifiably accessible that data analysis is treated as non-human subjects. Therefore no institutional review board approval and informed consent were required. We used the ICD-O-3 site codes C60.0 to C60.8 and histological codes 8,051–8,052 and 8,070–8,075 to identify SCCP patients. To develop the ideal ML model, several variables were obtained, including survival data, age at diagnosis, race, marital status at diagnosis, primary site, tumor size, tumor grade, surgical procedure, inguinal lymph node dissection (ILND), lymph-vascular invasion (LVI), TNM stage, radiotherapy data, and chemotherapy data. We grouped ethnicity into white, black and other; and marital status into married and other. An external validation set was constructed by collecting data on the same variables from our center based on the same criteria. A flow chart for patient selection from the SEER database is shown in Supplementary Figure S1. Early-stage ILNM was defined as N1 and N2 stage, i.e., unilateral or bilateral untreated ILNM.

### Statistical analysis

For continuous variables, Student's *t*-test was used for normally distributed data and the Mann Whitney *U*-test was used for non-normally distributed data. The chi-square test was used to analyze categorical data. Categorical variables were one-hot-encoded before incorporated into the ML algorithms.The odds ratio (OR) with 95% confidence intervals (CI) was calculated using univariate and multivariate logistic regression analysis. Only two-sided *p*-values <0.05 were considered to indicate statistical significance. We used five different ML algorithms to analyze our data: LR, XGB, RF, SVM, and kNN. The model with the highest average AUC was considered as the best algorithm. Furthermore, the ML-based model was tuned to avoid overfitting, and the accuracy of the algorithm was tested using the ten-fold cross-validation method. Detailed packages used in the development of our ML models including XGB 1.2.1, lightGBM 3.2.1, and sklearn 0.22.1. In the RF algorithm, the number of decision trees was set to 100, the maximum tree depth was set to 10, and the maximum number of leaf nodes was set to 50. In the kNN algorithm, the number of leaves was set to 30, and the number of nearest neighbors was set to 5. R 4.1.2 (https://www.r-project.org/), Python 3.10 (https://www.python.org/), and SEER*Stat (https://seer.cancer.gov/seers tat/) were used in this study.

## Results

### Patient characteristics

**Figure 1:**
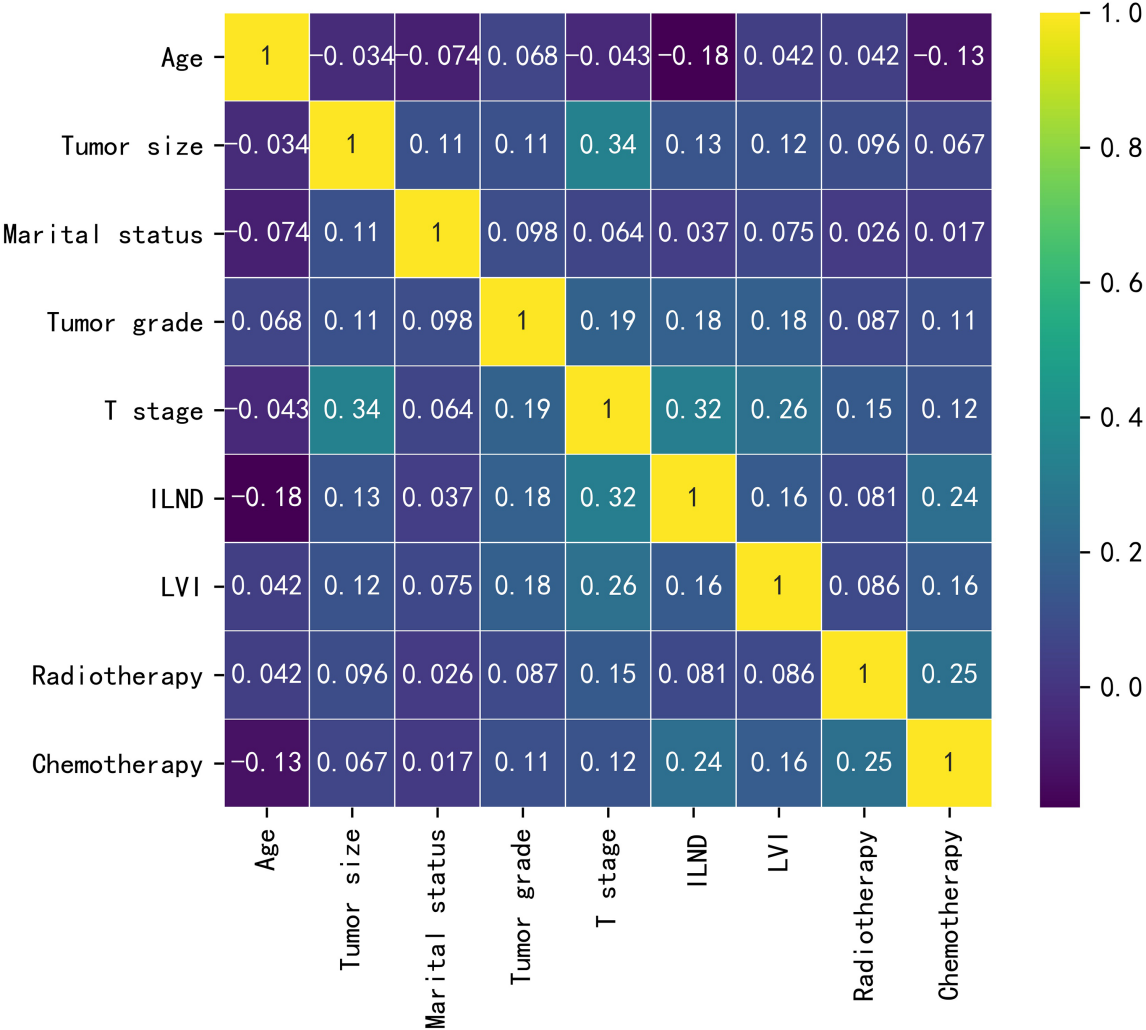
The results of Spearman correlation analysis between all the variables. The heat map shows the correlation between SCCP patients’ clinical and pathological features.

Baseline data for the training cohort and external validation cohort are provided in Table 1. In the training cohort, the variables with *p* values <0.05 were age, tumor size, marital status, tumor grade, T-stage, ILND, LVI, radiotherapy, and chemotherapy. Patients who developed early-stage ILNM were younger compared to those with stage N0 ILNM; had undergone ILND; and had larger tumor size, poorer grade, and higher T-stage. Further, a higher proportion of patients with early-stage ILNM had LVI and were receiving radiotherapy/chemotherapy. No statistically significant differences were found in race or primary site. The correlations between the variables chosen as predictors were analyzed and visualized by a heatmap using Spearman's rank correlation coefficient (Figure 1).

**Table 1:** Baseline characteristics of SCCP patients in the SEER database.

Variables	Level	Training cohort			External validation cohort		
* *		N0 (*n* = 892)	N1–N2 (*n* = 164)	*p*-value	N0 (*n* = 62)	N1–N2 (*n* = 12)	*p*-value
Age, year [median (IQR)]	NA	67.000 [57.000,75.000]	64.000 [52.000,73.000]	**0.003**	64.000 [59.000,74.000]	67.000 [51.000,75.000]	**0.809**
Tumor Size, mm [median (IQR)]	NA	27.000 [18.000,39.000]	35.000 [25.000,45.000]	**<0.001**	32.000 [25.000,40.000]	40.000 [30.000,50.000]	**0.161**
Race, *n* (%)	White	763 (85.538)	137 (83.537)	**0.552**	/	/	**/**
	Black	78 (8.744)	14 (8.537)		/	/	
	Other/Unknown	51 (5.717)	13 (7.927)		62 (100.000)	12 (100.000)	
Marital status, *n* (%)	Married	562 (63.004)	84 (51.220)	**0.004**	60 (96.774)	9 (75.000)	**0.006**
	Other status	330 (36.996)	80 (48.780)		2 (3.226)	3 (25.000)	
Primary site, *n* (%)	Prepuce	187 (20.964)	23 (14.024)	**0.138**	6 (9.677)	0 (0.000)	**/**
	Glans penis	565 (63.341)	109 (66.463)		25 (40.323)	4 (33.333)	
	Body of penis	72 (8.072)	14 (8.537)		28 (45.161)	6 (50.000)	
	Overlapping lesion of penis	68 (7.623)	18 (10.976)		3 (4.839)	2 (16.667)	
Tumor grade, *n* (%)	Well differentiated	300 (33.632)	15 (9.146)	**<0.001**	35 (56.452)	2 (16.667)	**0.003**
	Moderately differentiated	438 (49.103)	89 (54.268)		19 (30.645)	3 (25.000)	
	Poorly differentiated	150 (16.816)	59 (35.976)		6 (9.677)	6 (50.000)	
	Undifferentiated/Anaplastic	4 (0.448)	1 (0.610)		2 (3.226)	1 (8.333)	
T stage, *n* (%)	Ta-T1	506 (56.726)	36 (21.951)	**<0.001**	44 (70.968)	2 (16.667)	**/**
	T2	254 (28.475)	70 (42.683)		15 (24.194)	4 (33.333)	
	T3	128 (14.350)	55 (33.537)		3 (4.839)	4 (33.333)	
	T4	4 (0.448)	3 (1.829)		0 (0.000)	2 (16.667)	
ILND, *n* (%)	No/Biopsy only	755 (84.641)	44 (26.829)	**<0.001**	57 (91.935)	8 (66.667)	**0.014**
	Yes	137 (15.359)	120 (73.171)		5 (8.065)	4 (33.333)	
LVI, *n* (%)	Absent/Unknown	824 (92.377)	123 (75.000)	**<0.001**	58 (93.548)	4 (33.333)	**<0.001**
	Present	68 (7.623)	41 (25.000)		4 (6.452)	8 (66.667)	
Radiotherapy, *n* (%)	No/Unknown	853 (95.628)	127 (77.439)	**<0.001**	52 (83.871)	8 (66.667)	**0.164**
	Yes	39 (4.372)	37 (22.561)		10 (16.129)	4 (33.333)	
Chemotherapy, *n* (%)	No/Unknown	870 (97.534)	112 (68.293)	**<0.001**	58 (93.548)	9 (75.000)	**0.044**
	Yes	22 (2.466)	52 (31.707)		4 (6.452)	3 (25.000)	

ILND, Inguinal lymph node dissection; LVI, Lymph-vascular invasion, Other status including single, divorced, separated, divorced and unknown.

### Survival analysis

**Figure 2:**
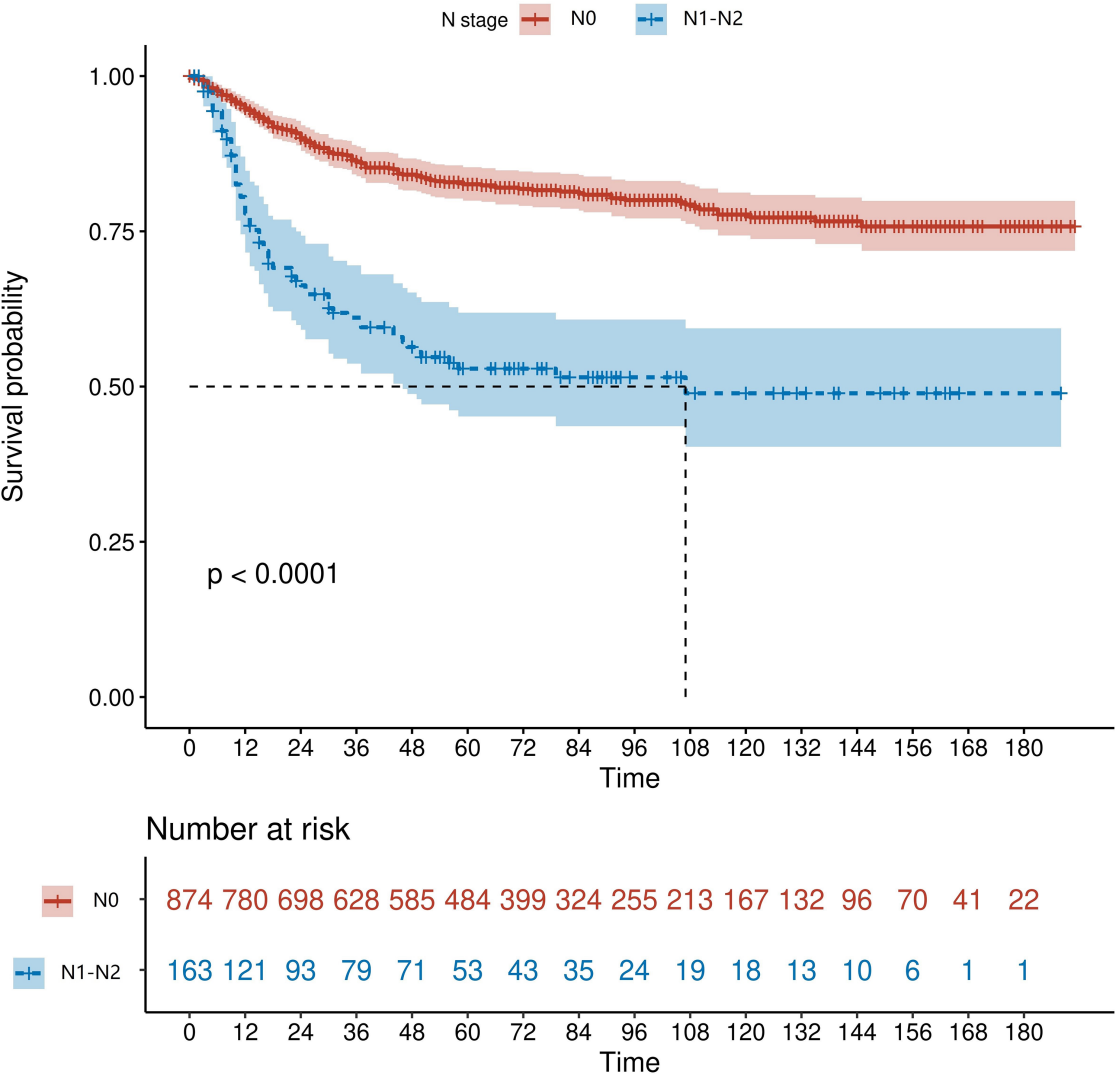
Kaplan-Meier curve of cancer-specific survival in SCCP patients.

We retrieved patients' survival data from the SEER database. Cancer-specific survival (CSS) was considered as the endpoint, and Kaplan-Meier survival analysis was used to estimate the survival. As shown in Figure 2, patients with early-stage ILNM had significantly worse CSS (*p* < 0.0001).

### Univariate and multivariate logistic regression analyses

As illustrated in Table 2, univariate logistic regression analysis showed that age, marital status, tumor grade, T-stage, ILND, LVI, radiotherapy, and chemotherapy were all significantly associated with the occurrence of early-stage ILNM in the overall population (*p* < 0.05). In multivariable logistic regression analysis, the factors with statistical significance included tumor grade (*p* = 0.001), T stage (*p* = 0.05), ILND (OR = 11.044, 95% CI = 6.984–17.846, *p* < 0.001), radiotherapy (OR = 4.64, 95% CI = 2.361–9.145, *p* < 0.001), and chemotherapy (OR = 9.612, 95% CI = 4.971–19.138, *p* < 0.001).

**Table 2:** Univariable and multivariate logistic regression analysis of the training cohort for predicting ILNM risk.

Variables	Level	Univariate OR	95%CI	*p*-value	Multivariate OR	95%CI	*p*-value
Age	NA	0.98	[0.967,0.992]	**0.001**	0.997	[0.98,1.014]	0.704
Tumor size	NA	1.002	[1.000,1.004]	0.083	/	/	/
Race	White	Ref		0.555	/	/	/
	Black	1	[0.550,1.816]	0.999	/	/	/
	Other/Unknown	1.42	[0.752,2.680]	0.28	/	/	/
Marital status	Married	Ref		**0.005**	Ref		0.08
	Other status	1.622	[1.160,2.267]		1.481	[0.953,2.304]	
Primary site	Prepuce	Ref		0.144	/	/	/
	Glans penis	1.569	[0.971,2.533]	0.066	/	/	/
	Body of penis	1.581	[0.771,3.241]	0.211	/	/	/
	Overlapping lesion of penis	2.152	[1.094,4.233]	0.026	/	/	/
Tumor grade	Well differentiated	Ref		**<0.001**	Ref		**0.001**
	Moderately differentiated	4.064	[2.306,7.160]	<0.001	3.057	[1.577,6.339]	0.002
	Poorly differentiated	7.867	[4.319,14.329]	<0.001	4.523	[2.197,9.83]	<0.001
	Undifferentiated/Anaplastic	5	[0.526,47.525]	0.161	8.396	[0.277,105.622]	0.15
T stage	Ta-T1	Ref		**<0.001**	Ref		**0.05**
	T2	3.874	[2.522,5.951]	<0.001	1.651	[0.955,2.872]	0.073
	T3	6.039	[3.802,9.594]	<0.001	2.199	[1.21,4.008]	0.01
	T4	10.542	[2.272,48.910]	0.003	4.64	[0.461,41.547]	0.183
ILND	No/Biopsy only	Ref		**<0.001**	Ref		**<0.001**
	Yes	15.03	[10.172,22.209]		11.044	[6.984,17.846]	
LVI	Absent/Unknown	Ref		**<0.001**	Ref		0.078
	Present	4.039	[2.624,6.218]		1.689	[0.937,3.016]	
Radiotherapy	No/Unknown	Ref		**<0.001**	Ref		**<0.001**
	Yes	6.372	[3.916,10.369]		4.64	[2.361,9.145]	
Chemotherapy	No/Unknown	Ref		**<0.001**	Ref		**<0.001**
	Yes	18.36	[10.743,31.378]		9.612	[4.971,19.138]	

OR, odds ratio; CI, confidence intervals; ILND, Inguinal lymph node dissection; LVI, Lymph-vascular Invasion, Other status including single, divorced, separated, divorced and unknown.

### Performance of ML algorithms

**Figure 3:**
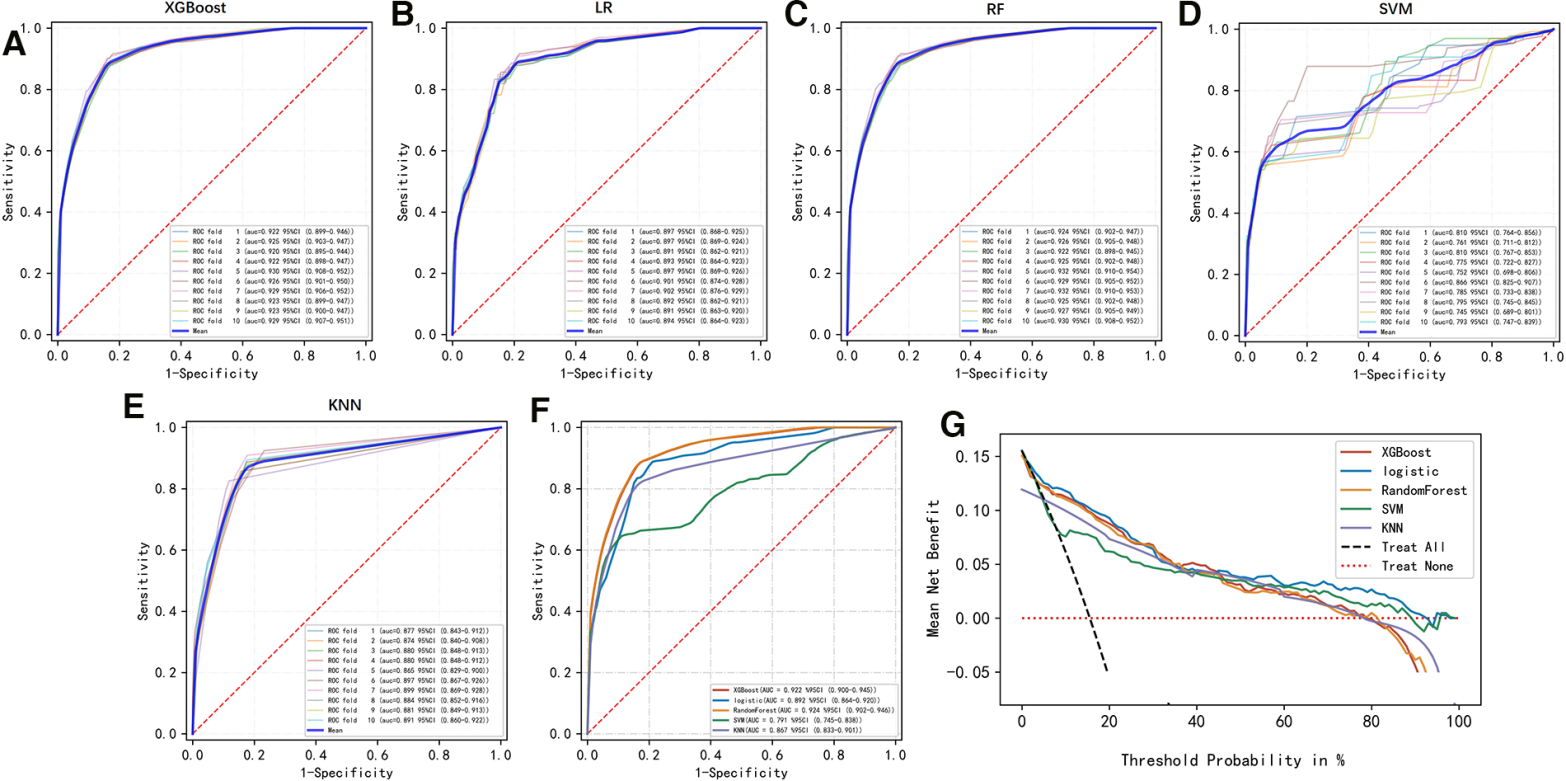
(**A–F**) Ten-fold cross ROC curves of five ML models in the training cohort. LR, logistic regression; XGB, eXtreme gradient boosting; RF, random forest; SVM, support vector machine; KNN, k-nearest neighbor. (**G**) Decision curve analysis graph showing the net benefit against threshold probabilities based on decisions from model outputs. The curves referred to as “All” represent the prediction that all the patients would progress to ILNM, and the curves referred to as “None” represent the prediction that no patients were ILNM.

**Figure 4:**
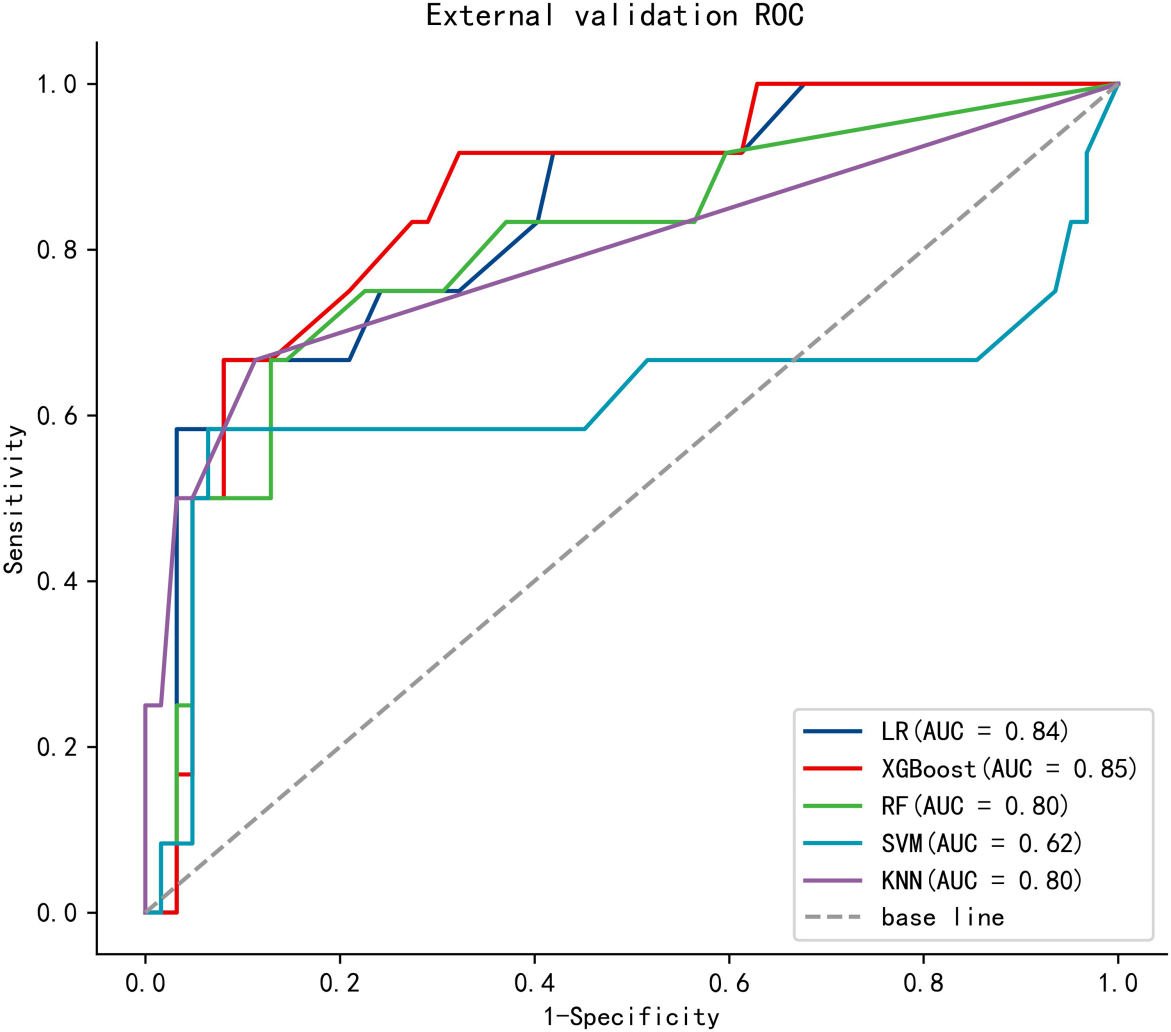
The ROC curve of five models in the external validation cohort.

To compare the predictive efficiency of the five ML algorithm models, ten-fold cross validation was applied in this study (Figure 3). Both the RF model (AUC = 0.924, 95% CI = 0.902–0.946) and the XGB model (AUC = 0.922, 95% CI = 0.900–0.945) performed well in the prediction of early-stage ILNM risk. The decision curve analysis of models was also subsequently constructed. The confusion matrix for ten-fold cross validation of training cohort is shown in Table 3. The learning curves of models in the training cohort are shown in Supplementary Figure S2. For the external validation cohort, as shown in Figure 4, the XGB model showed the best performance among the five algorithms according to receiver operating characteristic curve analysis (AUC = 0.853, 95% CI = 0.743–0.964). Since the XGB model was found to be stable and efficient in both the training and validation groups, we finally chose the XGB model (accuracy = 0.848, sensitivity = 0.887, specificity = 0.837 in the training cohort) as the final prediction model. Our ML algorithm model can be embedded in a web calculator or applet to allow clinicians to assess the risk of ILNM in patients with SCCP.

**Table 3:** Confusion matrix for ten-fold cross validation of training cohort.

	Accuracy	Recall	Precision	F1-Measure
XGB	0.867	0.867	0.86	0.857
LR	0.883	0.883	0.871	0.867
RF	0.863	0.863	0.853	0.852
SVM	0.873	0.873	0.859	0.851
kNN	0.869	0.869	0.861	0.861

### Relative importance of variables

**Figure 5:**
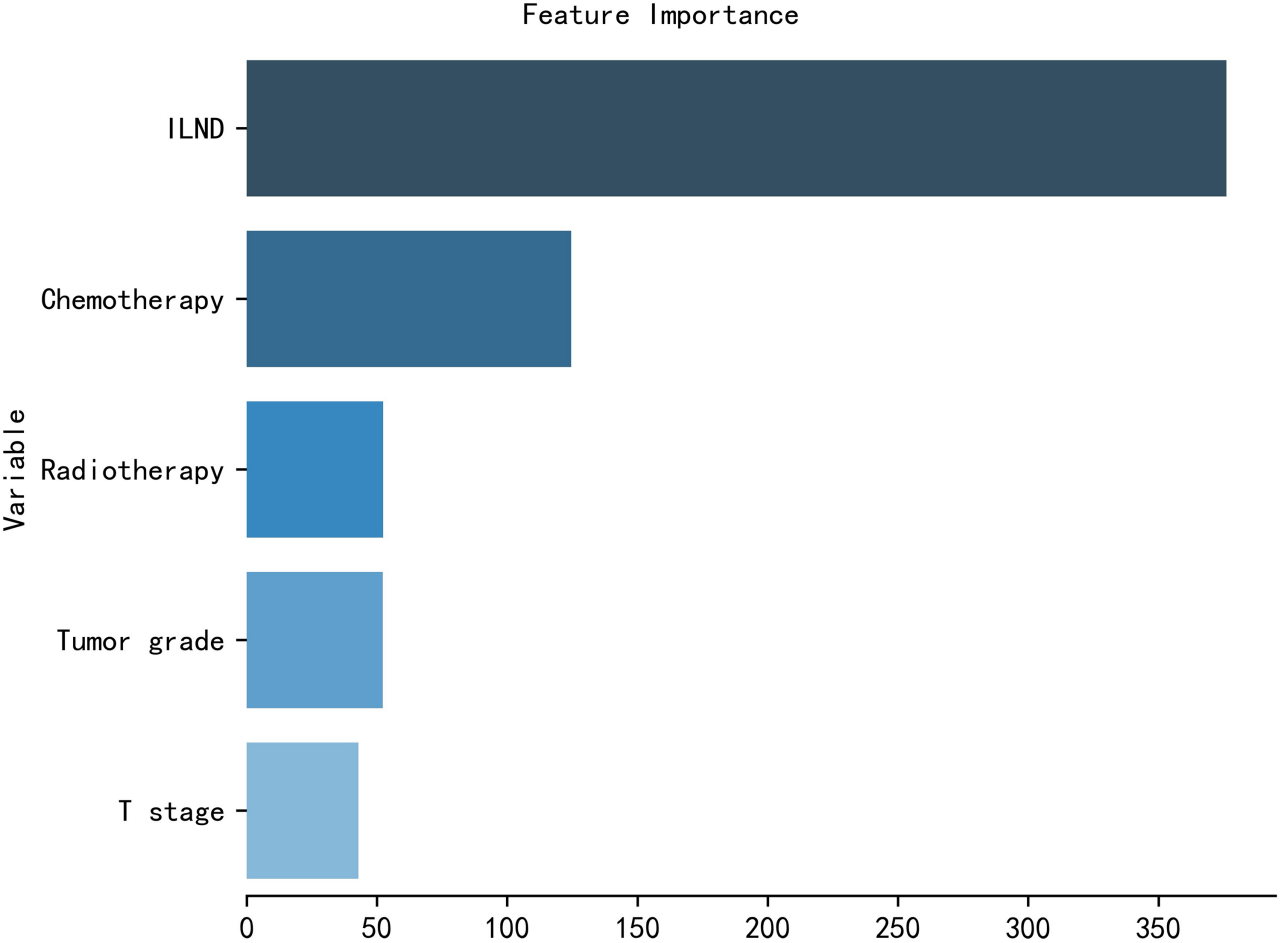
The XGB model was used to calculate the importance of each feature. The bar chart depicts the relative significance of the variables.

The importance of patient clinical features in the XGB model is shown in Figure 5, and is listed here in descending order of importance: ILND, chemotherapy, radiotherapy, tumor grade, and T stage.

## Discussion

The occurrence of ILNM in SCCP is associated with poor patient prognosis. ILND is the most important procedure for the prevention and treatment of ILNM (29). However, surgeons must consider the balance between the survival benefit to the patient and the high incidence of postoperative complications (30). The use of dynamic anterior node biopsy has been advocated to avoid unnecessary ILND, but the risk of false-negative results remains unavoidable (31), thus threatening the postoperative quality of patient survival. Therefore, early and accurate prediction of LNM is imminent. Previous studies on the SEER database suggest that age at diagnosis, tumor grade, tumor size, T stage, primary site, and LVI may be predictors of ILNM. However, these studies did not take into account the postoperative adjuvant therapy received by the patients. The models based on these variables had AUC of 0.776 and 0.795, respectively (20, 21). The advent of ML algorithms has made it possible to improve model performance.

In this study, we included SCCP patients no distant metastases and non-N3 stage, and developed an accurate predictive model for predicting the risk of early-stage ILNM based on multiple clinical and pathological indicators. Our aim is to screen the SCCP population with a high risk of ILNM for more careful perioperative management of this group of patients, with the ultimate goal of improving patient prognosis. To the best of our knowledge, this is the first model based on a variety of ML algorithms to predict early-stage ILNM with big data and performing external validation. This study included 1,056 cases of SCCP from the SEER database for model establishment and 74 cases from an independent institution in China for external validation. We adopted five ML methods: LR, XGB, RF, SVM, and KNN. By integrating the findings related to the effectiveness and stability of the models in the training and external validation groups, we finally identified XGB as the best prediction model algorithm for early-stage ILNM risk prediction. In the training cohort, the XGB algorithm model (AUC = 0.922, 95% CI = 0.900–0.945) showed superior performance and also performed well in the external validation. The model can be embedded as a web calculator or applied by way of a mobile terminal application. By entering the values or classification results of each indicator, the individual risk of progression can be calculated and, for patients at high risk, more aggressive physical examination, imaging, adjuvant therapy or even surgery should be performed postoperatively.

In both the baseline analysis and the univariate regression, a lower proportion of married than unmarried individuals had early-stage ILNM. This might partly be attributable to the fact that married people tend to have better economic conditions and personal hygiene. In addition, having physical intimacy with a partner increases the likelihood of detecting masses in the groin area and visiting a hospital at an earlier stage of the disease. In our study, the site of the primary tumor did not show a significant association with ILNM risk, but this is not consistent with the findings of previous studies (21). Because delayed intervention can adversely affect the survival of patients with PC diagnosed with lymph node involvement, treatment guidelines recommend lymph node dissection. Therefore, management of regional lymph nodes is very important for patient survival (32). Our findings indicate the importance of ILND for the detection of early-stage ILNM. LVI is defined as infiltration of tumor cells in the lymphatic or hematologic system. Previous studies have shown that LVI is significantly associated with lymph node metastasis and distant metastasis (33). Recently, there is evidence that the presence of LVI is an important risk factor for occult micrometastases in patients with penile cancer and affects the overall survival of patients (34). The correlation of risk with T stage and nuclear grade observed in the present study is in line with previous literature (21, 34). Meanwhile, patients with indications for radiation and chemotherapy, have a higher risk of developing early ILNM, based on clinician judgment.

Our study has certain limitations. First, the number of patients used for external validation at our center is small, and we hope to further improve it at a later stage through multi-center collaboration. Second, ML algorithms are inconvenient for use in clinical practice, and we hope to develop an online calculator app of our XGB model for clinicians in the future. In addition, there are also some limitations to the data in the SEER database. The lack of data on immunohistochemistry, genomics, patient physical indicators, underlying diseases, and hematological indicators reduces the possibility of improving the accuracy of the model, and we hope to remedy this through the establishment of a multicenter database. In addition, the practical value of the model was determined in a predominantly Caucasian database, so its applicability in other regions (including China) is unclear due to the inevitable differences in ethnicity and treatment levels in different countries/regions. Finally, the indications for ILND or adjuvant therapy vary from one medical center to another, so there may be some errors in its practical application. Nevertheless, our study yielded encouraging results that ML algorithms appear to have greater efficacy potential for early ILNM risk prediction in patients with SCCP compared to traditional logistic regression analysis.

## Conclusion

We have built a precise, big data-based ML model for predicting early-stage ILNM in patients with SCCP. External validation proved that our novel model has excellent predictive accuracy and clinical utility. Therefore, in the future, it may guide clinicians' decisions and improve the long-term prognosis of patients.

## Data Availability

The raw data supporting the conclusions of this article will be made available by the authors, without undue reservation.
