# Supplementary figures and images for: A machine learning-based model for predicting the risk of early-stage inguinal lymph node metastases in patients with squamous cell carcinoma of the penis
Source: Front Surg. 2023 Mar 17;10:1095545. doi: 10.3389/fsurg.2023.1095545 (PMC10063794; doi:10.3389/fsurg.2023.1095545)

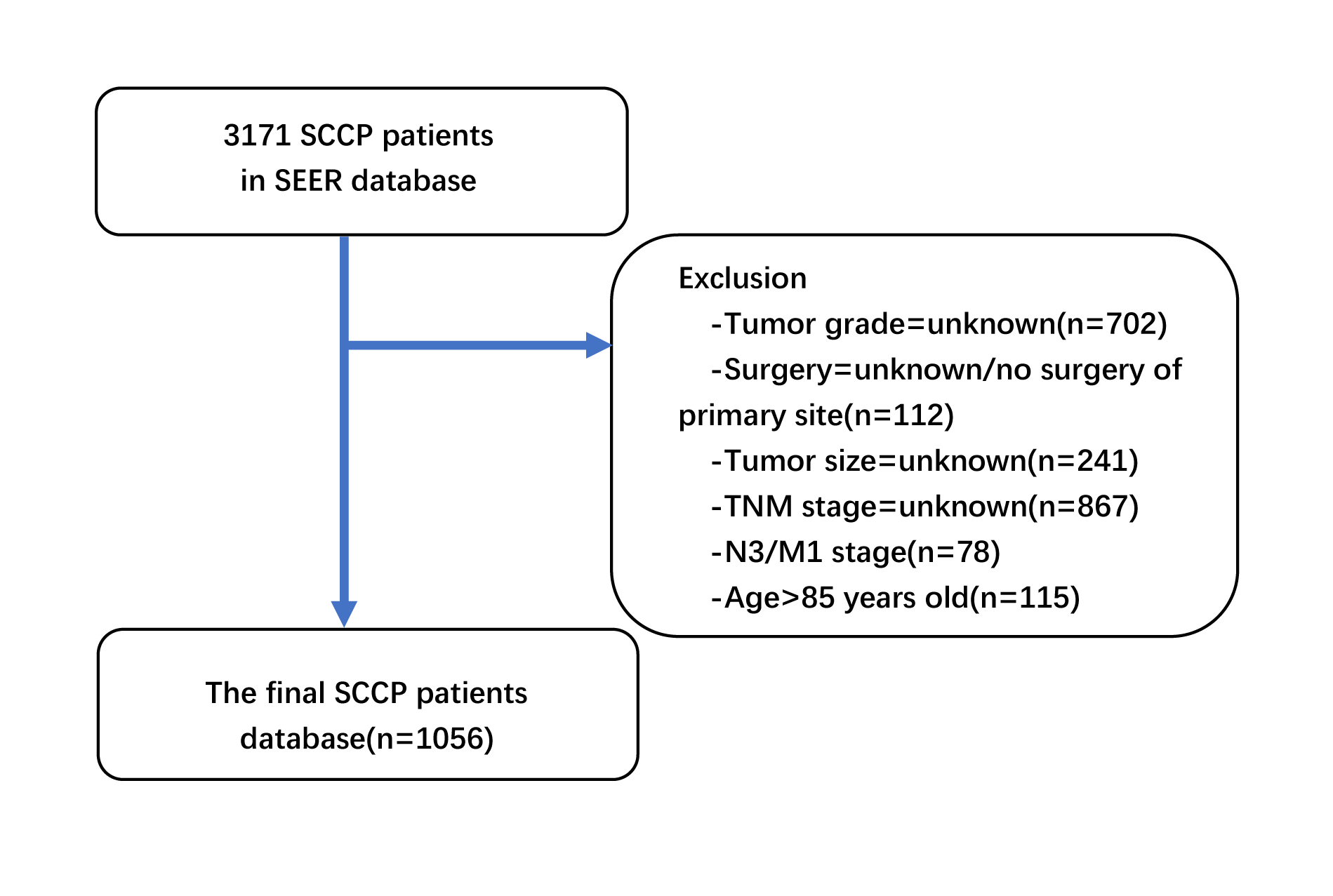

Supplement: Supplementary file 3 [file Image1.tif]

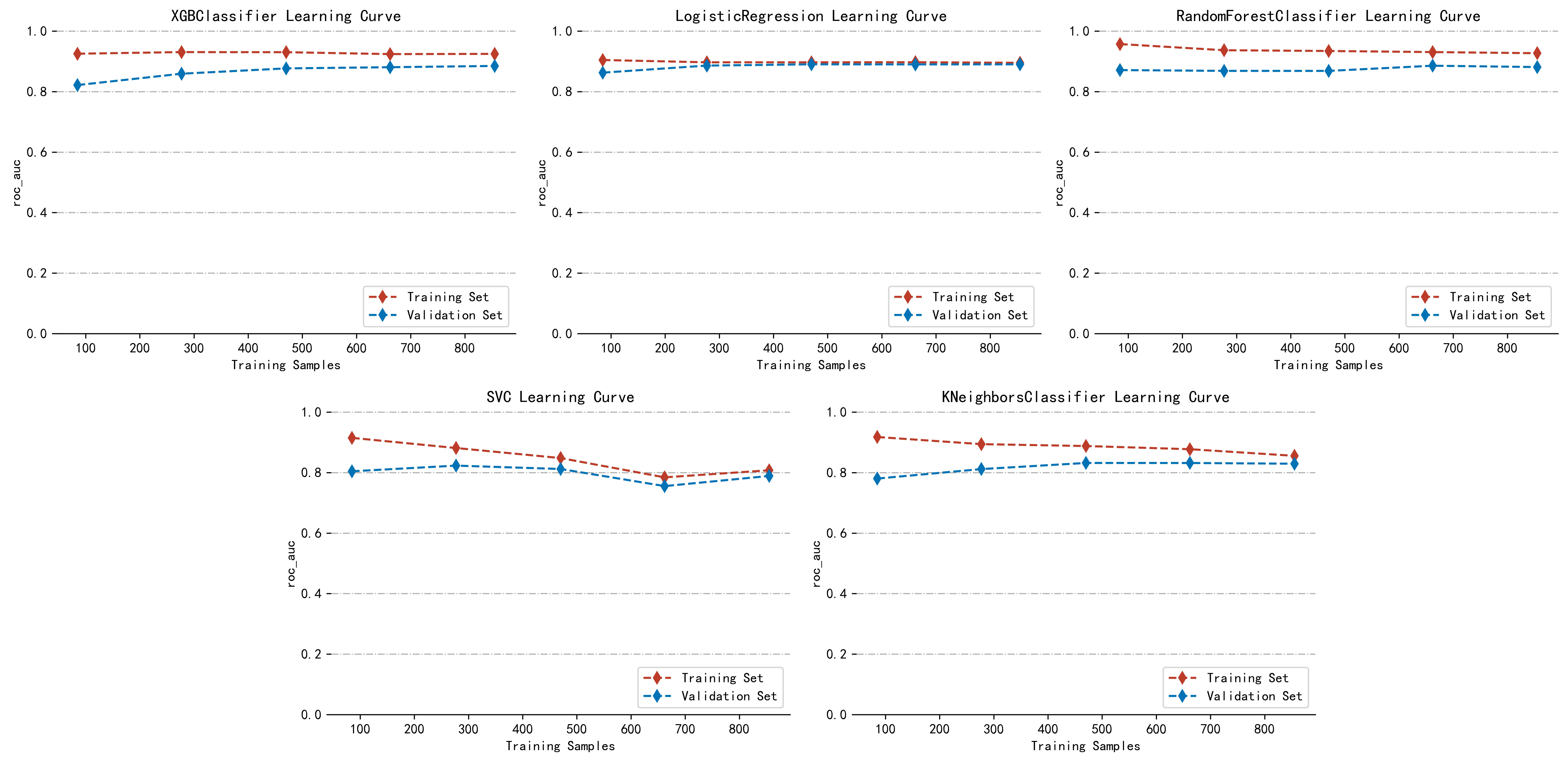

Supplement: Supplementary file 4 [file Image2.tif]
